# Exploring the participant experience in controlled human infection model (CHIM) trials: A modified grounded theory study

**DOI:** 10.1371/journal.pone.0328378

**Published:** 2025-08-06

**Authors:** Anna G. Mack, Donna M. Halperin, Bailey M. Selig, Brian R. Condran, Scott A. Halperin

**Affiliations:** 1 Canadian Center for Vaccinology, Dalhousie University, IWK Health, Nova Scotia Health, Halifax, Nova Scotia, Canada; 2 Rankin School of Nursing, St. Francis Xavier University, Antigonish, Nova Scotia, Canada; 3 Nova Scotia Health Learning Institute for Healthcare Providers, Nova Scotia Health, Halifax, Nova Scotia, Canada; KEMRI-Wellcome Trust Research Programme, KENYA

## Abstract

**Background:**

Controlled human infection model (CHIM) trials are useful tools for accelerating vaccine development through the deliberate infection of healthy volunteers in a controlled environment to study the course of disease in humans. CHIM trials may pose physical, psychological, and social harms to participants. Participants are subjected to long inpatient stays and daily sample collection, which may be uncomfortable. Very little is known about the CHIM trial participant experience and decision-making process.

**Methods:**

We conducted a modified grounded theory study, embedded within the Canadian Center for Vaccinology (CCfV)’s *Bordetella pertussis* CHIM trial. Twenty six participants engaged in semi-structured interviews at four time periods throughout their year-long involvement in the CHIM trial, using a constant comparative approach of simultaneous data collection and analysis. This process continued until saturation was reached and an emerging theoretical model was constructed depicting the CHIM trial participant experience over time.

**Results:**

The emergent conceptual model centered around the core category “A Trusting Partnership”, depicting the evolution of the participant-researcher relationship throughout the experience. Establishing trust was critical during the decision-making phase, facilitated through transparent researcher-participant communication and consideration of past experiences and values. Supporting the trusting partnership during the inpatient isolation phase was facilitated through ongoing researcher-participant engagement and emotional support. During outpatient participation, maintaining the trusting partnership was achieved through effective communication, consistent follow-up care, and recognition of participants’ contributions. Quality improvement (QI) recommendations were identified across all phases of CHIM trial participation.

**Conclusions:**

Researcher-participant trust is an integral component of the CHIM trial participant experience. QI recommendations should be taken into consideration when planning and coordinating future CHIM trials.

## Background

Controlled human infection model (CHIM) trials (also known as human challenge studies, or challenge trials) are powerful tools to accelerate vaccine development, using the intentional infection of healthy participants with a disease-causing pathogen to study the pathogenesis and clinical course of disease in humans [1]. CHIM trials are integral to vaccine development against many harmful pathogens, such as malaria infection [2]. CHIM trials can pose potential physical, psychological, and societal harms to volunteers, which must be justified by the research’s scientific and social value and effectively managed according to ethical guidelines [3]. For instance, participants may experience uncomfortable symptoms of infectious diseases and may have to endure extended inpatient isolation periods [4]. The success of CHIM trials is highly dependent on the availability of eligible and willing volunteers. There are no direct health benefits provided by CHIM trials to participants; therefore, thorough consideration of risks and benefits of healthy volunteers is required to ensure participation is ethically sound [5].

Historically, the prevailing consensus among researchers has been that healthy volunteers in clinical research trials are solely motivated by financial incentives [6,7]. In this regard, the researcher-participant relationship has been equated by some to a business transaction [8]. This gives rise to concerns surrounding undue influence, wherein participants assume risks beyond their understanding due to overly enticing financial incentives [4]. While any expense incurred related to study participation should be reimbursed, any additional compensation offered to cover risks associated with participation may be difficult to quantify and can contribute to undue inducement [5]. Participants most vulnerable to such exploitation include individuals of low socioeconomic status; however, exclusion based upon economic status risks being discriminatory [4,9,10]. These ethical concerns surrounding clinical trial participation warrant further research to more deeply understand the motivations and experiences of CHIM trial participants.

Emerging research exploring the motivations of CHIM participants have found the decision-making process to be more complex than originally thought. CHIM trial participants have been found to articulate a nuanced array of motivations when engaging in quantitative surveys, citing altruistic motives and a desire for new experiences alongside financial incentives [9,11,12]. While quantitative data are useful to quantify the motivations for CHIM participants, they fail to describe how participants make meaning out of their experiences and motivations. To date, three qualitative studies have explored the experiences and perspectives of CHIM trials participants, reporting complex decision-making processes and outlining participants’ understanding of study burdens [11,13,14]. While these findings serve as an important first step, they do not consider the temporal aspect of the CHIM trial participant experience. Furthermore, since these studies were conducted within malaria CHIM trials, further research is needed to explore and compare participant experiences in other types of CHIM trials. Additionally, these studies were conducted within the USA and Kenya, therefore reflecting the experiences and perspectives of CHIM trial participants within their respective contexts. Our qualitative study aimed to expand upon the existing literature by developing an emerging theoretical model exploring the decision-making processes and experiences of Canadian *Bordetella pertussis* CHIM trial participants over time.

## Methods

This qualitative modified grounded theory research took place as a sub-study embedded within the Canadian Center for Vaccinology’s (CCfV) *Bordetella pertussis* CHIM trial. *B. pertussis*, also known as whooping cough, is one of the most prevalent vaccine-preventable diseases [15]. A CHIM trial would allow investigation of *B. pertussis* pathogenesis in humans as a step towards developing a next generation vaccine. Healthy, eligible volunteers were inoculated, or “challenged”, with a dose of *B. pertussis* and monitored on the CCfV Challenge Unit, located at the IWK hospital in Halifax Nova Scotia, in single-bed isolation rooms for 16–21 days, pending positive *B. pertussis* infection and subsequent antibiotic administration. During this time, participants underwent routine sample collection and clinical testing, including blood draws, nasal swabs, and nasopharyngeal aspirates. Participants were compensated at a rate of CAD 350 per day spent in isolation. Participants continued to be monitored and attend regular follow-up visits for a year post-challenge.

This sub-study followed a modified grounded theory approach to develop a theoretical understanding of the CHIM trial participant experience rather than description [16]. Our modified grounded theory approach utilized a pragmatic philosophical perspective to inductively generate a process-based theory depicting CHIM trial participant experiences and perspectives over time [17]. Symbolic interactionism was employed through in-depth participant-led interviews to explore how participants make meaning out of their environment and experiences [18], alongside a patient-centered lens to center participants as experts in these experiences [19].

All 79 participants enrolled in the *B. pertussis* CHIM trial were invited to participate in the sub-study via flyers upon admission to the CCfV Challenge Unit from July 8^th^, 2022, to May 27^th^, 2024. Some participants were approached directly by a member of the study team upon admission to the Challenge Unit and invited to participate in the sub-study. Interested participants were contacted to schedule initial interviews to discuss their decision-making process, experience to-date, and collect demographic data. Interviews were held with 26 CHIM trial participants at up to four time points throughout their year-long involvement in the CHIM study (2–4-, 10–14-, 56-, and 365-days post-challenge, respectively). These interviews were semi-structured, guided by a set of open-ended pre-determined questions, but largely participant-led. Participants were encouraged to lead conversations and explore topics of personal relevance related to their experiences in the CHIM trial. The longitudinal approach to data collection functioned to enhance the credibility of our qualitative findings by capturing the dynamic nature of participants’ experiences through repeated engagement [20]. Interviews were held either in-person, via phone, or via secure videoconferencing platforms (Zoom) at the participant’s convenience. Interviews spanned 30–60 minutes in length. Not all participants completed all four interviews. A total of 68 interviews were conducted, with an average of 2.6 interviews per participants. Participants were compensated for their time (CAD 50 for the first interview, plus CAD 25 for each subsequent interview completed).

**Fig 1:**
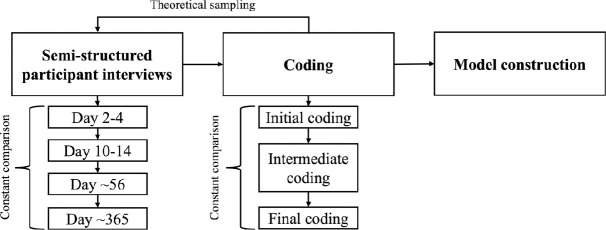
Iterative Data Collection and Analysis Process. Constant comparison of transcripts within and between participants assisted with theoretical sampling of interview questions, modifying the semi-structured interview guide with subsequent interviews. An iterative coding process by multiple coders led to the construction of our emerging theoretical model.

All interviews were audio recorded and transcribed by a professional transcriptionist for analysis. In keeping with our modified grounded theory approach, the constant comparative method was used throughout data collection and analysis to facilitate comparisons within and across the data (i.e., within each individual participants over time and between participants) [18]. Interviews followed flexible semi-structured interview guides (Attachments 1 and 2 in S1 File) which were continuously adapted throughout the research process; each interview yielded new questions for data collection and analysis to aide in the development of our emerging theoretical model [21]. The use of field notes during interviews and memo writing in-between interviews assisted with this theoretical sampling process [14,22]. Fig 1 presents the cyclical data collection and analysis process.

Transcripts were initially coded by three independent researchers (AM, BS, BC) in NVivo 14 to identify emerging patterns, followed by collaborative intermediate coding to group open codes into broader categories. This inter-researcher triangulation functioned to establish credibility of findings and minimize bias [23]. Final coding was used to draw connections between categories and identify our core category, ensuring that all concepts were closely interrelated and generate our emerging theoretical model [24]. This analytic process occurred alongside data collection until there was inter-researcher agreement (AM, BS, DH, SH) that theoretical saturation had been obtained. Visual diagrams were used throughout the research process to represent the relationships between the identified categories, while a detailed audit trail recorded the research process to reflect the grounding of the final theory in the data [23].

### Ethical considerations

This study received research ethics board approval from both the IWK and St. Francis Xavier University. All transcripts were checked for accuracy and any participant-identifiers were removed to maintain confidentiality. Researchers received infection prevention and control training by the challenge unit nursing staff prior to data collection. Proper personal protective equipment was worn, and safety precautions were maintained, during all in-person interviews to ensure researcher and participant safety. Verbal consent was sought from participants prior to initial interviews and were audio recorded.

## Results

Of the 79 CHIM trial participants enrolled in the CCfV’s *B. pertussis* CHIM trial, 26 participants were recruited. Participants for CCfV’s CHIM trial consisted of healthy adults between 18–40 years of age; the mean age of participants in our sample was 29.88 years. None of the participants had previously participated in a CHIM trial; only two participants had previously participated in other kinds of clinical trials. Demographic information can be found in Table 1.

**Table 1:** Demographic Information (N* *= 26 Participants).

Characteristics	Frequency (%)
*Age*	
18-25	8 (30.77)
26-33	10 (38.46)
34-40	8 (30.77)
*Sex assigned at birth*	
Male	12 (46.15)
Female	14 (53.85)
*Gender*	
Male	11 (42.31)
Female	11 (42.31)
Non-binary/Agender/Transgender	4 (15.39)
*Race*	
White^a^	23 (88.46)
*Student*	
Yes	5 (19.23)
No	21 (80.77)
*Education level*	
High school or college diploma/certificate/technical training	4 (15.39)
Some university	5 (19.23)
Undergraduate university degree	14 (53.85)
Master’s degree	3 (11.54)
*Employment status*	
Unemployed, not looking for work	5 (19.23)
Unemployed, looking for work	8 (30.77)
Employed	13 (50)
*Annual household income in CAD, before tax* ^b^	
Up to 20,000	8 (30.77)
20,001-60,000	5 (19.23)
Over 60,001	9 (34.61)
Not reported	4 (15.38)

^a^Small cells were collapsed to maintain anonymity.

^b^1 CAD = 0.70 USD.

**Fig 2:**
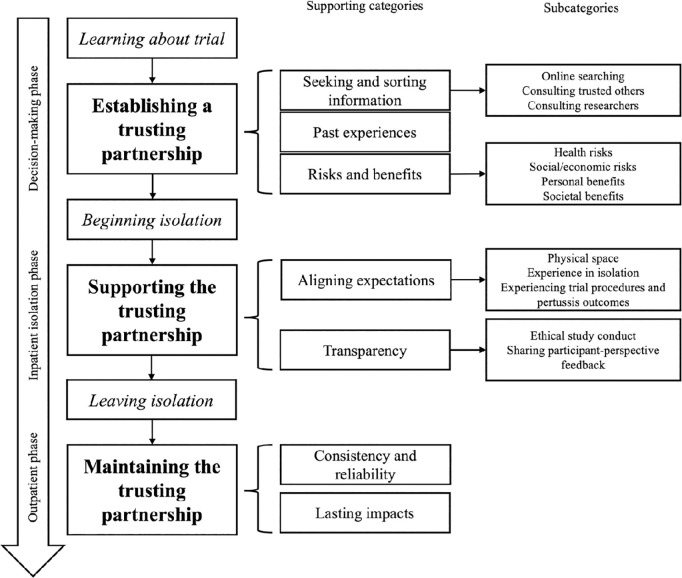
Emergent Conceptual Model for the Participant Experience in CHIM Trials. This model depicts the trajectory of the CHIM trial participant experience in three phases, with each phase corresponding to a stage in the trusting partnership between participants and researchers: (1) establishing; (2) supporting; and (3) maintaining. Supporting categories and subcategories are presented to reflect the most prominent themes for participants at each phase of the study.

Through rigorous data collection and analysis, *a trusting partnership* emerged as the core category that encapsulates the essence of the CHIM trial participant experience. This emergent conceptual model traces the trajectory of participants’ experiences over time, encompassing the initial decision-making process, the inpatient isolation stay, and the outpatient follow-up visits. The model highlights the dynamic and evolving nature of trust and partnership between participants and researchers (including both Challenge Unit clinical staff and investigators), revealing key factors that can influence participants’ decisions and experiences at each stage of the trial. The analysis of data revealed three phases that describe the CHIM trial participant experience over time: (1) establishing a trusting partnership (during the decision-making phase, where trust is built through transparent researcher-participant communication, consideration of past experiences and values, and comprehensive informed consent processes); (2) supporting the trusting partnership (during the inpatient isolation phase, where trust is sustained by ongoing engagement, consistent care, and emotional support); and (3) maintaining the trusting partnership (during the outpatient follow-up phase, where trust is upheld through effective communication, consistent follow-up care, and recognition of participants’ contributions). Across all three phases, clear communication between researchers and participants is an integral component of a successful trusting partnership; communication functions to provide necessary information, aide in decision-making, respond to emotions, establish expectations, and foster relationships [25]. Therefore, clear communication is interwoven throughout the CHIM trial participant experience as both an antecedent and an ongoing facilitator of a trusting partnership. Seven categories were identified across all three phases to reflect the most important themes for participants at each phase (1) Information seeking and validation; (2) Past experiences; (3) Risks and benefits; (4) Aligning expectations; (5) Transparency; (6) Consistency and reliability; and (7) Lasting impacts. Subcategories were constructed to capture the multiple dimensions of the processes within categories. Our emergent conceptual model is presented in Fig 2.

### Establishing a trusting partnership: Pondering the path to partnership

Participants cannot make the decision to participate in the CHIM trial before understanding and establishing trust in the researchers’ rationale, methods, and legitimacy. Initial discovery of the trial sparked an internal and interpersonal discourse to determine facts about, opinions on, and experiences participating in CHIM trials; participants drew on their own past experiences, perspectives, and beliefs and sought out new information from various sources. They also weighed the risks of the study, real and/or perceived, against the overall benefits, societal and/or personal, they expected to receive. The final decision to participate in the trial was rooted in trust participants had established in the researchers, resulting in the formation of a mutually beneficial researcher-participant partnership.

### Seeking and sorting information

Participants reported hearing about the trial from a variety of sources, such as online media, word of mouth spread, and community advertisements. In particular, the tagline included on the trial’s recruitment materials, “Are you up for a challenge?”, was found to be enticing by many participants. Some participants who discovered the trial online reported skepticism regarding the legitimacy of the study, whereas those who heard from other sources, such as from the researchers themselves, did not share these concerns.

*“Honestly, I applied a bit on a whim. I just happened to come across some online info about the trial and thought that it seemed like it might be interesting. I put my name in. I didn’t expect to hear back at all, honestly… anything that has incoming money online seems like it’s a 50/50 chance of, if not worse, of being legitimate versus being somebody trying to steal personal data.” (P16, Male, Age range 26-33, Employed fulltime, Interview 1)*

*“At my university there was a nurse there who, I think, was involved with the study and she was handing out pamphlets when we came to get our vaccines at this little clinic they had set up and so you could go up, you could talk to the nurse and so I asked her some questions about the study, what it would involve, when I might be able to participate and then she sort of sent me the email and I registered online.” (P12, Male, Age range 18-25, Unemployed, Interview 1)*

Most participants were unfamiliar with either CHIM trials, *B. pertussis,* or both. Upon initial discovery of the trial, participants sought out more information to aide them in making an informed decision regarding their ability to trust the researchers. Sources included the Internet, trusted others within participants’ social circles, and the researchers themselves.

*“I would say I definitely did a little bit of digging. Like having little knowledge of whooping cough beforehand. I knew that it was at least fairly serious and once I did the digging and it’s been very clear with all the paperwork and talking to all the nurses and what not that it is a very safe environment in here which has definitely made me feel more comfortable doing it.” (P20, Male, Age range 26-33, Employed fulltime, Interview 1)*

Participants conducted independent research online to develop a better understanding of what participation in the CHIM trial would entail, and how it would impact them. Sources mostly included the CCfV’s website and news articles about CHIM trials. Anecdotal reports from past CHIM participants were found to be especially helpful in the decision-making process, as one participant stated:

*“…so, I went online and looked up other people’s experiences and found what I could and pretty quickly I had a good grasp of what it would be like… Even just hearing what people did to keep themselves busy, inspired a little bit of confidence in signing up for it.” (P17, Male, Age range 18-25, Unemployed, Interview 1)*

Some participants identified the potential effects of *B. pertussis* inoculation as the most important information they obtained prior to making the decision. For others, aspects of the inpatient isolation were the most pertinent. Participants overall seemed satisfied with the extent of the information provided by the CCfV’s website.

*“I remember kind of looking into what are the long terms effects of getting this disease because that’s obviously the biggest concern if I’m getting inoculated with something and I did my own research on that within the website…” (P10, Female, Age range 26-33, Employed fulltime, Interview 1)*

*“I was concerned about the isolation which, then I read the website and it’s like oh, okay, everything’s kind of accounted for and it doesn’t seem so bad.” (P13, Female, Age range 18-25, Student, Unemployed, Interview 3)*

Almost every participant expressed seeking the opinions of others when making the decision to trust the researchers and participate in the CHIM trial, including family, friends, and physicians. These discussions served to assist participants in making sense of the potential risks and benefits of participation in the trial and reinforcing their trust in the researchers. For some, these trusted individuals were crucial in their decision-making process, while others placed less emphasis on external opinions.

*“So, I did have a conversation with my brother and another friend just talking about if I should take it what that would look like and they were talking about the pros and cons…” (P21, Female, Age range 34-40, Employed fulltime, Interview 1)*

*“…and I called my doctor and told him about it and asked if he had any concerns…” (P22, Female, Age range 26-33, Employed fulltime, Interview 1)*

*“The opinions of the people around me didn’t really matter that much. If my parents had been dead-set, I would have maybe reconsidered but I didn’t think they would be too wary.” (P13, Female, Age range 18-25, Student, Unemployed, Interview 1)*

While some participants reported positive responses from their social circles upon expressing their interest in the CHIM trial, others were received with some hesitancy and skepticism. Participants reported reiterating the study’s safety when convincing hesitant friends and family.

*“I would say my parents were somewhat hesitant at first until they read through it. Some people I knew just weren’t really interested in doing anything like that. They didn’t like the idea of having a live bacteria, but yeah, mostly positive.” (P12, Male, Age range 18-25, Unemployed, Interview 1)*

Most participants reported reaching out to the researchers directly to have any concerns addressed prior to making the decision. The pre-trial screening visits provided participants with an opportunity to have any concerns alleviated before their challenge date, including concerns about the inoculation, the physical space, or the amenities offered.

*“Yeah, so I sort talked with the staff and they talked about how the dosage schedule worked and how they would find a dose or try to find a dose that wouldn’t elicit symptoms ideally even if some people would have those symptoms and then also learning about the antibiotics… they would do five days antibiotics and that would clear your system and you’d be good to go after.” (P12, Male, Age range 18-25, Unemployed, Interview 1)*

The opportunity to ask the researchers clarifying questions was an important step for participants in providing informed consent as it offered them a space to obtain all the necessary information to make a fully informed decision. There was an appreciation for the staff’s empathetic approach to these conversations, as one participant explained:

*“Anything that I didn’t understand was explained to me. It just felt very human, like it wasn’t so cold like, yes or no survey type questions. It was like, we’re trying to understand how you can help us and we can help you.” (P13, Female, Age range 18-25, Student, Unemployed, Interview 3)*

Some participants disclosed having pre-existing relationships with members of the study team. This was found to facilitate an added sense of comfortability and trust in the researchers, thus contributing to the decision to participate.

*“No, [study team member] talked to me about it and I know [study team member] wouldn’t be trying to sell something that wasn’t safe, especially to her friends so I didn’t really have any health concerns.” (P15, Female, Age range 26-33, Unemployed, Interview 1)*

### Past experiences

In addition to considering recently acquired information about the CHIM trial, participants also drew on their own past experiences when establishing trust in the researchers. Previous individual isolation experiences, such as mandated COVID-19 quarantines, functioned to shape participants’ perceptions of the inpatient stay, as well as their own capabilities to live on the challenge unit, as stated by this participant:

*“Yeah, I probably still would have done it before but just going through Covid now, I realize I can handle it pretty well. I might have been more nervous if I hadn’t had prior experience and just being away from social contact for as long as I have been.” (P2, Male, Age range 18-25, Student, Unemployed, Interview 1)*

Past experiences accessing healthcare services or participating in research, both within the IWK hospital or the CCfV and beyond, factored into participants’ ability to trust the researchers. Generally, negative experiences were identified as deterrents to participation, whereas positive experiences were facilitators. One participant recounted having had a positive experience participating in a research study conducted by the CCfV in the past, and explained how this instilled a level of confidence and trust in the researchers:

*“I had done some previous work with the vaccinology center, some volunteering… it was a very positive experience. Everybody was very kind, very patient, very efficient and were very eager to make me comfortable throughout the whole experience.” (P7, Female, Age range 34-40, Employed fulltime, Interview 1)*

Somewhat conversely, one participant expressed a desire to participate despite resonating with historical trauma within the research and medical fields as a Black individual. They explained how these negative past experiences encouraged them to participate in research to diversify study samples and improve the relevancy of research fundings to Black populations:

*“…but also, it was more like I know a lot of diverse individuals because of histories and trauma within the medical field do not willingly put themselves forward to participate in research studies and I’m just like, you know I may just be one drop in the bucket, I’m like I can at least do some variation to that.” (P21, Female, Age range 34-40, Employed fulltime, Interview 1)*

### Risks versus benefits

All participants reported perceiving some type of benefit from their participation in the CHIM trial. Participants perceived societal benefits, personal benefits, or both. Participants assessed what they expected to gain out of participation and weighed this against the potential risks, including physical/mental health risks and economic/social risks.

Personal benefits were oftentimes perceived by participants alongside the societal benefits of participation; however, each participant ascribed importance to these possible benefits based on their own individual experiences, preferences, and values. While all participants expressed their thoughts on the monetary compensation provided for their participation, some participants viewed this as a primary motivator, while others considered it as a pleasant, but ultimately unnecessary, bonus.

*“I’d say my primary motivation for pretty much everything is monetary. Money is always good.” (P17, Male, Age range 18-25, Unemployed, Interview 2)*

*“…even if there wasn’t compensation, I probably would still have been interested in doing it anyway, but the fact that there is, is nice.” (P8, Agender, Age range 26-33, Unemployed, Interview 1)*

A relationship between economic/employment status and financial gain was noted in the data when examining compensation as a primary motivator. Overall, participants who disclosed having low financial stability, or participants who were students or unemployed, seemed to hold the financial compensation in higher value than others. One participant exhibited this relationship when explaining how their initial decision-making process was influenced by their employment status at that time:

*“Initially when I first heard about it, it was the money. I’ve got a better job so that’s not the motivator anymore.” (P9, Female, Age range 34-40, Employed fulltime, Interview 1)*

Other personal benefits aside from financial compensation were noted. Some participants saw the CHIM trial as an opportunity to experience something novel and were attracted by the notion of using the time in isolation to work on personal projects, challenge themselves, or simply relax.

*“…as well as I feel like this whole experience is kind of, it’s very unique and might not be able to have something like this again, so to be able to say that I did it is definitely something that’s pretty cool to me.” (P20, Male, Age range 26-33, Employed fulltime, Interview 1)*

*“…and I thought, I thought it would be kind of fun. Get away from the house for a little bit, sort of like a little vacation in a hotel except more needles.” (P2, Male, Age range 18-25, Student, Unemployed, Interview 1)*

A few participants made mention of access to medical screenings as a personal benefit of participation. The screening process allowed participants to receive a full medical examination to rule out any potential medical conditions that may exclude individuals from participation. This was especially valuable to those participants who were without family doctors, or to those who would otherwise struggle to obtain these screening tests.

*“It was kind of interesting even if I hadn’t qualified to be able to just go see how I’m doing because you know there’s not exactly a lot of free time in doctors’ schedules for checkups on random adults to see how you’re doing as a human. You go to the doctor when you have an issue so it was kind of nice to be able to go get this kind of screening and I would have been happy to get it even if I didn’t qualify.” (P6, Female, Age range 26-33, Employed part-time, Interview 3)*

*“…you get a full medical which nobody ever really thinks of when they talk about this. Get better healthcare and right now I don’t have a family doctor so that’s great… but I’ve also lost my family doctor in that time so the motivation has changed from when I initially started this process to actually getting here.” (P9, Female, Age range 34-40, Employed fulltime, Interview 1)*

Although not all participants cited altruism as a primary motivator for enrollment, every participant acknowledged the societal benefits of participating in the CHIM trial. Some participants considered research participation as a kind of civic duty, whereas others benefited from the feeling of giving back and contributing to their communities.

*“I feel like the least I can do is offer up my own understanding to help other people receive the care that they need and improve their quality of life. Part of the basis of living in a society is that we all help one another…” (P6, Female, Age range 26-33, Employed part-time, Interview 1)*

For some participants, the societal benefits of participation held more weight in their decision-making process than personal benefits. Many altruistically motivated participants reported a history of altruistic acts, including blood donation, prior research participation, or surrogacy. Proximity of the disease under study to oneself, such as personal connections to pertussis or other respiratory diseases, was shown to augment the perceived importance of these societal benefits. One participant explained the importance of these societal benefits in relation to their child:

*“Well, I have a daughter. I’m having another kid, so pertussis is important because that’s the thing that you’re like go get vaccinated to everyone. You want to touch the baby, you have to go get a shot, that kind of stuff.” (P4, Male, Age range 34-40, Unemployed, Interview 3)*

All participants in our sample were vaccine-accepting. Many were eager about the prospect of contributing to the development of new vaccines and positively impacting the health of others, as this participant explained:

*“…eventually this should help develop a better vaccine to protect babies against whooping cough so when my mom was like oh my gosh, why would you do this? I’m like, why wouldn’t I?” (P3, Female, Age range 34-40, Employed fulltime, Interview 2)*

Participants highlighted some of the health risks they perceived when making the decision to participate in the CHIM trial, including the physical health risks of *B. pertussis* infection along with the mental health risks associated with the lengthy inpatient isolation period. The consensus among our sample was that the physical health risk of *B. pertussis* infection for healthy adult volunteers was relatively low, which contributed to their trust in the researchers and willingness to participate. Some participants further believed the health risks of participation to be relatively low given their own personal histories with illness, self-reporting “strong immune systems”. This led to a belief that they would not develop serious illness from *B. pertussis* infection.

*“…so, with pertussis, with whooping cough where I’m in that age range where it’s not particularly dangerous to me, I’m being actively monitored, there’s access to immediate treatment. I considered it a very low risk but depending on what you’re signing up for I expect that there could be higher degrees of risk… I can’t imagine any challenge trial, at least in Canada, being signed off on without being relatively safe.” (P16, Male, Age range 26-33, Employed fulltime, Interview 3)*

*“Again, I’m not very educated in healthcare in general but at the very least you know it’s like, whooping cough is like, it’s old. It’s something I’ve heard about for a long time so just through exposure I’m confident that we can treat it in adults and it’s not going to have significant consequences on my health once I’m out of here.” (P17, Male, Age range 18-25, Unemployed, Interview 1)*

Some participants perceived the mental health risks of participation to be higher than the risks of *B. pertussis* inoculation; however, ultimately decided to participate despite acknowledging these risks.

*“Yeah, I really didn’t have any concern about the risks like physically or medically. It was more about my mental health sort of declining because of the isolation…” (P22, Female, Age range 26-33, Employed fulltime, Interview 1)*

The logistics of living in isolation for 16–21 days posed some economic and social risks for participants. Being away from one’s partner, mitigating work responsibilities, missing out on opportunities, delegating household work, and impacts of the timing of the trial were reported to be challenging to navigate. Many participants in our sample worked remotely or were granted permission from their employer to work remotely for the duration of the trial. Being able to maintain a source of income while participating in the trial, despite receiving the study compensation, was reported to be a necessity by some. As one participant stated:

*“If I didn’t work from home, I wouldn’t have been able to do this. I wouldn’t have taken three/four weeks off work to do this. It wouldn’t make any sense, so it was, for sure, a huge factor.” (P10, Female, Age range 26-33, Employed fulltime, Interview 1)*

Some participants who planned to work remotely while participating in the CHIM trial disclosed their participation to their workplace and reported positive and supportive responses from their supervisors and coworkers, suggesting minimal social risk. Others, however, chose to conceal participation from their workplace, either out of fear of negative responses, accusations of abusing work from home privileges, or simply a desire for privacy.

*“It’s not necessarily a reason, it’s more like I don’t like, that’s not to say I don’t like folks in my business but there was nothing really to say… they just know I’m working from home, they don’t know why.” (P21, Female, Age range 34-40, Employed fulltime, Interview 1)*

The economic risks of participation were found to be lesser for participants who were unemployed. Some of the students in our sample opted to participate at the end of their semesters when they would normally be looking for summer jobs, using the monetary compensation to supplement employment.

*“For me as a student, just coming out of university, I don’t really have a lot to do right now. I didn’t have a job lined up, so it was sort of a good fit for me. It would be different, I suppose, if it was somebody who was coming in from work, but I didn’t have to give up work or really anything aside from just my time.” (P12, Male, Age range 18-25, Unemployed, Interview 1)*

The timing of the trial in relation to life responsibilities was found to be a critical factor in the decision-making process by most participants. For some students, for instance, navigating school responsibilities around the timing of the trial was found to be challenging. One participant recounted their struggles planning for the CHIM trial without putting their education at risk:

*“Yeah, the timing really had to work out. I was worried because I actually had an exam like the week before this happened, it was in person… The biggest issue isn’t staying, it’s finding the time to even do it.” (P2, Male, Age range 18-25, Student, Unemployed, Interview 2)*

### Supporting the trusting partnership: Immersion in the trial environment

Once trust in the researchers had been established and the decision to participate had been made, participants were immersed in the inpatient portion of the CHIM study. Supporting the newly established trusting partnership during this stage is critical; a strong, supportive researcher-participant partnership was found to have the capacity to facilitate participant retention throughout the inpatient and outpatient stages of the study. This was achieved when participants were satisfied with the conditions of their stay, perceiving an alignment between pre-trial expectations and the reality of the inpatient isolation stay, and participants perceived the researchers to be honest, open, and dependable.

#### Aligning expectations.

Participants were found to set certain personal expectations for the inpatient period during their pre-trial preparations, including expectations about the physical environment, how they might spend their time in isolation, and the trial procedures and outcomes (i.e., the development of *B. pertussis* infection and/or symptoms). Alignment of these expectations with inpatient experiences can support participants’ trust in the researchers; conversely, when there were unexpected surprises during their experience, some participants reported feeling deceived by the researchers. Most participants in our sample felt as if the trial overall proceeded as expected or better; the strong alignment between expectations and reality strengthened their trust and opinions of the researchers.

*“…It didn’t impact my day-to-day as much as I thought that it would when I was deciding to or not to sign up so I’m really glad that I did.” (P7, Female, Age range 34-40, Employed fulltime, Interview 4)*

*“I would say it’s beyond what I was expecting which has been really great.” (P6, Female, Age range 26-33, Employed part-time, Interview 2)*

The physical environment was found to have a profound impact on participants’ experiences during the inpatient isolation period. Factors such as the size of the room, the privacy it allowed for, and the amenities offered were some of the topics participants discussed. Most participants deemed the rooms to be adequately sized to continue their regular routines, with ample storage for their possessions. Many participants spoke to the importance of personalizing the space, bringing in items from home to instill a sense of comfort and familiarity. Misalignment between participants’ individual expectations and their interpretation of the physical space upon admittance to the challenge unit posed challenges, as expressed by these participants:

*“The most difficult it was just in the first two days, and I think it was just more about aligning the expectations. Whenever I talked to my friend previously, she had mentioned somebody was able to bring games and play games so my expectation was to be able to bring my gaming system and it would just hook up to the TV, but it didn’t work because the TVs didn’t work.” (P4, Male, Age range 34-40, Unemployed, Interview 2)*

*“Yeah, [the blinds] cannot be pulled up. It makes it feel like a prison and had I known I, I don’t know if I would agree to do the study and because they did all the interviews and testing in that far room, that far room doesn’t do that I felt misled.” (P11, Trans female, Age range 34-40, Unemployed, Interview 2)*

Seeing the space as their own and being able to carry out regular routines was important for many participants. Interruptions to the space and participants’ sense of privacy were highlighted as a challenge. Participants appreciated the consideration of the nursing staff when planning to enter the rooms; however, they recounted instances where privacy was not maintained or when their routines were disrupted to accommodate the study needs:

*“I think it feels extra disruptive to have people come in to your space sort of unplanned and the nurses are really good about being like I’m coming in at 9 or I’m coming in at this time and then just like have people come in a bit randomly, especially sometimes you’d be in the bathroom and they’d just come in and like, so maybe just, I don’t know if it’s IWK but having more notice that this is happening.” (P10, Female, Age range 26-33, Employed fulltime, Interview 3)*

*“…like you’re told ahead of time that no one will disturb your sleep… I know we have to have certain things to the lab by a certain time too so that’s totally understandable but there’s a couple of nurses that are like, I guess inpatient or just want to get going and so they’ll go into the little room there and turn the lights on and then let the door slam on their way out in like a very subtle way of waking you up I guess and then like 10 minutes later I’ll get an intercom (noise) hey, are you up yet? and it’s like obviously yes, you just slammed the door in the hallway.” (P3, Female, Age range 34-40, Employed fulltime, Interview 2)*

Given that the CCfV Challenge Unit is located at the IWK Children’s Hospital, sensory aspects of living in a hospital environment were also noted. Participants recounted certain small, seemingly mundane aspects of the environment and described the significant impacts they had on their experience in isolation, including the temperature of the showers, hallway lights, noise from the air vents, and the sound of the call bell. Participants recognized that many of these aspects were unavoidable in the hospital setting, referring to them as quirks rather than challenges.

*“I guess negatives if you will which really aren’t negative, it’s just more like quirks, things that are a little bit different here then at home. If I want to take a shower that is so hot that I look like a lobster when I get out because I’ve pretty well scalded myself, I can’t do that here because it’s a children’s hospital.” (P3, Female, Age range 34-40, Employed fulltime, Interview 2)*

*“In here, you know, for example the vent in the ceiling is going 24/7 at varying degrees of sound and in my own home if there was a vent on I could turn it off which is like a thing I miss being able to do.” (P6, Female, Age range 26-33, Employed part-time, Interview 2)*

*“The noise is, I mean, just like any other noise you become very accustomed to certain things when you’re subjected to it for a long time. The buzzer, like when you call people into your room, it didn’t, at first, I thought wow, that’s loud. If you think about it, it has to be loud because somebody needs to hear it in an area but at first, I was very aware of it. Especially at night things like that.” (P7, Female, Age range 34-40, Employed fulltime, Interview 3)*

Despite the unavoidable nature of these environmental elements, some participants believed many of these issues might have been mitigated had they been more informed prior to beginning isolation, allowing them to better prepare prior to their challenge day.

*“I was a bit aghast initially at all the disposable food packaging. I didn’t realize that would be the case necessarily so that sort of horrified me just because single use plastic and stuff and I know it’s a hospital, but I mean there’s nothing really I could have brought. I guess I could have brought a metal spoon and fork and knife, but I don’t know, I don’t know if they would have and like don’t include utensils in my order. I don’t know if that was an option.” (P22, Female, Age range 26-33, Employed fulltime, Interview 2)*

*“…the fact that they did the interview in the room that doesn’t have the blinds in it felt like they were trying to hide something. At least I could have been prepared. I could have brought something in to kind of make the room feel bigger. I have a big like forestry tapestry that I could have hung up or something like that. I could have taken steps to mitigate forward had I known.” (P11, Trans female, Age range 34-40, Unemployed, Interview 2)*

The way participants intended to spend their time in isolation was found to be highly individualistic. Some planned to work remotely, whereas others planned to use the time on personal projects and hobbies. Some participants reported having passed the time in isolation exactly as expected, as one participant explained:

*“…so, it is kind of playing out the way I had expected that you can take the time to do what you want to do and if you don’t want to exercise that day, you don’t have to and if you don’t feel like reading that day, you don’t have to. If you want to sit all day and eat snacks and watch movies, absolutely.” (P3, Female, Age range 34-40, Employed fulltime, Interview 2)*

Conversely, some found their initial plans for the stay to be less feasible than anticipated. For instance, some participants who had plans to continue their schoolwork in isolation struggled to work and relax in the same space.

*“It’s something I noticed is now an issue because before, it happened the first week, but I was just [like], I’m in a new area, it was just messing me up. As it went on a second week and my second assignment, I was like this is just difficult to work in here.” (P2, Male, Age range 18-25, Student, Unemployed, Interview 2)*

Some participants were pleasantly surprised by the routine of life in isolation, stating that their full-time employment and sample collections filled most of their days, leaving little time for hobby-related activities and relaxation. A few participants even made mention to having increased productivity in isolation, attributed to a lack of distractions in comparison to their usual working environment.

*“I think when you first consider coming here before you’ve gotten here and part of me wondered, like I brought all these activities to do because I was thinking I was going to be so bored… but especially because I’ve been working my regular shifts, 8-hour days, that’s a big part of your day and then if you consider that in the morning you’ve got samples… by the time you get off work and you have vitals again at 7, your day is kind of full and then you just kind of have the time you would have at home.” (P7, Female, Age range 34-40, Employed fulltime, Interview 2)*

*“I think surprisingly I’ve felt more productive here than at home. I think also because I’m probably more of a socially active person at home so I have a social event at 6 p.m. so I couldn’t keep working, but here I can finish working or do a little bit extra work, so I have nothing else to do.” (P10, Female, Age range 26-33, Employed fulltime, Interview 2)*

Conversely, some participants who were unemployed relished the opportunity to be unproductive while in isolation, describing their experience as a welcomed break from responsibility:

*“I think the thing for me is that I don’t need to feel productive while I’m in here. It’s more of, that’s sort of what the break is for me. It’s not, um, like I haven’t been really focusing on the “what I’ve accomplished today” or, you know, how many steps I got or anything. I’m just in here so, that’s the accomplishment I guess.” (P13, Female, Age range 18-25, Student, Unemployed, Interview 2)*

Despite most participants having had prior isolation experience with the Covid-19 pandemic, some were surprised by the difficulty of voluntary isolation as opposed to mandatory quarantines. They recounted their struggles with the confinement, especially upon hearing of friends and family socializing and feeling left out, as one participant explained:

*“So, for me using those two experiences that I did have with the Covid world… I’m like okay my expectation is that I should be fine because I’ve had to isolate before and that was very manageable versus actually being in isolation, in a very specific context where I volunteered myself to do this… the first weekend I was there all my friends went out and they were all hanging out meanwhile I’m stuck in isolation and I can’t do anything about it and just not really, no matter how much you prepare for that you can’t control how you’re going to react to that…” (P5, Non-binary, Age range 26-33, Employed fulltime, Interview 1)*

Other participants struggled with being dependent on study staff for everyday needs; given that participants were confined to their rooms, they relied on staff to complete tasks like restocking supplies or doing laundry. Many participants confessed to feeling awkward or uncomfortable asking staff to do these tasks that they were physically capable of doing themselves.

*“I mean, I knew people were going to be coming in and doing stuff I just, yeah, like, I don’t think I anticipated feeling that weird because I hadn’t thought ahead about if I want a bag of chips. I have to press the button and ask someone who’s probably busy doing something else to stop what they’re doing and bring me a little fun size bag of potato chips for my snack.” (P3, Female, Age range 34-40, Employed fulltime, Interview 2)*

The participants were offered the use of an online Discord sever to facilitate discussions among their cohorts, offering them an opportunity to share their experiences with other participants and coordinate weekly take-out meals. The consensus in our sample was that the Discord sever was underused. It was suggested that this was due to a lack of understanding on how to access the server, a general lack of interest, or feelings of discomfort in conversing with strangers online. Some participants didn’t care to use the server, whereas others were excited to converse with other participants. One participant went as far as to state that the opportunity to meet new people was an initial motivating factor in their decision to participate and was disappointed with the Discord’s lack of use.

*“…one of the things that interested me when I read the original article is that they talked about Discord or having an online chat community with the other participants… I think that was one of the appealing things. I was like oh, I can meet other random people doing the same thing as me. I guess I’m a little bit disappointed that’s not super active.” (P10, Female, Age range 26-33, Employed fulltime, Interview 1)*

Daily sample collections serve to shape the CHIM participant experience. Participants undergo sample collections during screening visits, which enables them to set expectations for the inpatient testing procedures, such as the level of discomfort they can expect to endure. Certain sample collection procedures, such as the nasopharyngeal aspirates (NPAs), were reported by some participants to improve over time, whereas others, such as blood draws, were described as more uncomfortable with repetition.

*“For instance, the nasal wash or the NPA so those were definitely things that I had never really heard of before and so were definitely weird the first time doing it and a bit unexpected of what the sensation would be like but kind of by day three I was like alright I know exactly what to do here, we’re good to go.” (P24, Male, Age range 18-25, Student, Unemployed, Interview 1)*

*“I find the needles a little worse now. I don’t know if it’s not because I got poked so much in that one day but I do get a little more nervous now when I’m getting blood drawn then I was, it’s not as bad as when I first came in when I literally couldn’t even look to the side because I would see it but at the moment, I don’t know, maybe I’m just a little sore after all the needles.” (P2, Male, Age range 18-25, Student, Unemployed, Interview 2)*

Being prepared on what to expect day-to-day was found to be valuable for participants. Knowing the sample collection schedule allowed participants to mentally and physically prepare for various procedures; for instance, knowing which days were blood draw days prompted participants to drink more water to ensure easy venous access. One participant made mention to the detailed information packet provided to them at the beginning of the trial and described its usefulness in terms of sample collection:

*“Yeah, I refer to it almost every single night just to see the next day what sort of samples they’ll be taking, what sort of, pretty much what sort of samples they’ll be taking, what to prepare myself for the next day. Is it like getting blood taken so I should get my arms ready to be poked with a needle or am I getting my nasal cavities washed out with saline so make sure my nose is ready to be shot up.” (P24, Male, Age range 18-25, Student, Unemployed, Interview 1)*

Integrating sample collections into daily routines was found to be challenging for some participants. Participants were able to coordinate with the nursing staff to schedule sample collections around their individual responsibilities and preferences; however, certain extenuating circumstances, such as lab delays, made this difficult, as one participant explained:

*“Delays in the lab have caused interruptions before. It would have been easier if I had known about that in advance so I could kind of adjust my schedule more.” (P9, Female, Age range 34-40, Employed fulltime, Interview 2)*

The outcome of the pertussis challenge itself (i.e., positive or negative for *B. pertussis* infection) was found to impact participants’ experiences. Positive infection may induce symptoms and often requires participants to remain in isolation for longer. Participants were found to form expectations regarding the outcome of the challenge; some participants believed they would become symptomatic, whereas others felt as if they would not. These expectations were formed based on knowledge about the *B. pertussis* dosage they were receiving, as well as their own perceived susceptibility to illness.

*“It’s an interesting thing because it’s not blinded so I knew what my dose was, I know that’s, I don’t know if there’s like a reverse placebo, but I knew, I was pretty confident I guess that I wasn’t going to get symptoms.” (P1, Female, Age range 26-33, Student, Employed fulltime, Interview 2)*

*“Well for a while there I honestly thought I would be one of the asymptomatic people, just like not show any signs of being sick for myself or for the tests but, it was kinda a surprise honestly when I started coughing, I really wasn’t expecting it.” (P13, Female, Age range 18-25, Student, Unemployed, Interview 2)*

Disjoint between individual expectations and the actual challenge outcome were found to impact participants’ experiences. Participants who became unexpectedly symptomatic were faced with challenges regarding the increased duration of stay, such as arranging transport home and mitigating responsibilities.

*“In the practical sense I definitely would have had to renegotiate with my workplace to be out longer. I had planned it as 16-18 days it, including weekend days so I would have had to probably use sick time to be out… it would have been probably harder on my partner for managing the house and the dog and everything all alone and having my help back home being pushed that much further because that’s hard to do for that long…” (P6, Female, Age range 26-33, Employed part-time, Interview 3)*

#### Transparency.

Upon placing their trust in the researchers, participants expected full transparency from the study team during all procedures, including disclosing any deviations from the study protocol and providing rationales for all study decisions. Observing ethical study conduct through rigorous consent processes and having opportunities to provide feedback were found to be integral to participants’ perception of the researchers’ transparency. Breaches in transparency were found to impair researcher trust, risking participant withdrawal from the study.

It was important to participants to observe ethical study conduct throughout the inpatient stay. Participants recounted feeling reassured by the steps taken by the researchers to mitigate any potential exploitation of financially unstable participants, as one participant stated:

*“…someone said you have to have a home address to participate so avoids trying to ask people to participate when it may be their only option. It was important to me that it didn’t feel like they’re specifically trying to get low-income people.” (P10, Female, Age range 26-33, Employed fulltime, Interview 1)*

Ongoing consent-seeking before every study procedure was observed and appreciated by participants, serving to support participants’ trust in the researchers.

*“…I really appreciate a lot of things that are happening in this study like constant, informed consent, questions like being able to always opt out and that’s really, really important I think…” (P10, Female, Age range 26-33, Employed fulltime, Interview 1)*

*“…but the nurses were always like, how do you feel about this, is it okay if we do this today, before each one, not just before we started overall, and they were very good about checking in.” (P6, Female, Age range 26-33, Employed part-time, Interview 3)*

Overall, participants appreciated the empathetic nature of the researchers. Most participants reported feeling safe and comfortable throughout the isolation experience, attributed to the attentiveness of the staff.

*“…seeing the way that there should always be hypothetically empathetic and considerate researchers interacting with study subjects and awareness of people’s, of power in different situations and awareness of people’s wellbeing and it was really, I think, reassuring to be honest to get to see how that carried through and to experience it in such a nice way from all of the staff that I interacted with.” (P6, Female, Age range 26-33, Employed part-time, Interview 3)*

Some participants posed some ethical concerns regarding the financial compensation provided for study participation. Some believed the day-by-day nature of the compensation might compromise the study’s integrity by enticing financially motivated participants to deceive the researchers, while others thought additional compensation should be provided upon the development of a new vaccine which would be highly profitable to large pharmaceutical companies.

*“…I wouldn’t be surprised if people tried to fake it to stay longer just because it’s another $200 every day you’re here. I would not be surprised if people faking being sick to stay longer…” (P2, Male, Age range 18-25, Student, Unemployed, Interview 2)*

*“I was thinking about this and how when I was reading in the paperwork it’s like this might lead to a new vaccine… it would be lovely someday if participants in the study benefited when a vaccine actually came out because I know drug companies make a ton of money… because really if you think about how much people make being in these it’s less than minimum wage per hour and it’s your life and your time.” (P9, Female, Age range 34-40, Employed fulltime, Interview 1)*

Having the opportunity to provide feedback about various aspects of the study was found to facilitate a sense of transparency between participants and staff. Participants appreciated the open dialogue they had with the study staff:

*“...generally speaking, the staff seemed super attentive and concerned about just making sure they were getting feedback on stuff.” (P4, Male, Age range 34-40, Unemployed, Interview 3)*

*“They also have been quite considerate that it is like a space that I’m living in at the moment… this is the time space that we need to do this in, what works best for you? Let’s be considerate of one another and it’s been a dialogue which I really appreciated.” (P6, Female, Age range 26-33, Employed part-time, Interview 1)*

Strong professional relationships between participants and nursing staff were identified. Given that the nursing staff provided essential social interaction within the care setting, participants often experienced meaningful and supportive interactions with the nursing team while maintaining appropriate professional boundaries. This comfortability instilled a sense of ease in participants when it came to verbalizing any needs, as one participant stated:

*“...so, I did feel comfortable with the nursing staff which really helped me to vocalize the things that I did want or need.” (P5, Non-binary, Age range 26-33, Employed fulltime, Interview 1)*

Any discomforts related to the sample procedures, the physical environment, or the amenities provided were quickly resolved by staff, according to participants.

*“I mentioned that I was sore, and the staff immediately worked to find a solution and they have topical ointment to help with pain management and kind of just like, like what I said at the beginning that as soon as I mention a need or a concern immediately there was problem solving and working together and finding something that would mitigate that struggle.” (P6, Female, Age range 26-33, Employed part-time, Interview 2)*

*“I want them to have a pretty good read of like if I am uncomfortable with too many pokes in one day, I’m going to tell them or if I’m uncomfortable with some other aspect of the room or something about the food or other things, I’m trying to tell them…” (P1, Female, Age range 26-33, Student, Employed fulltime, Interview 2)*

While some participants had no trouble vocalizing concerns, considering it to be an integral component of the study, others struggled with feeling like an inconvenience to staff.

*“It’s just that I’ve been encouraged pretty consistently by just about everyone that I’ve touched on with the study to be open and honest where possible and to just kind of be forward with the experience that I’ve had so I didn’t feel badly about it. I just kind of looked at it as part of the study.” (P7, Female, Age range 34-40, Employed fulltime, Interview 1)*

*“I don’t want to be inconveniencing of having to move and feel like I’m complaining, but yesterday I was like no, I think I put up with this long enough that yesterday morning it was decided that we would just move rooms…” (P21, Female, Age range 34-40, Employed fulltime, Interview 2)*

### Maintaining the trusting partnership: Transitioning from inpatient to outpatient

The conclusion of the in-patient isolation stay does not signify the end of participation; follow-up visits continue for a year post-challenge as participants transition back to their regular lives. Maintaining the trusting partnership throughout outpatient participation was found to be vital for participant retention. This was achieved when participants observed consistency during all study procedures and perceived the researchers to be reliable. Lasting impacts of the CHIM trial on participants’ lives post-isolation were also found to influence the maintenance of the trusting partnership, either positively or negatively.

#### Consistency and reliability.

The outpatient needs of the CHIM trial consisted of follow-up phone calls and appointments at the IWK. Balancing these outpatient requirements with everyday responsibilities was noted as relatively easy; participants were able to schedule calls and appointments with a degree of flexibility to accommodate their own schedules and preferences.

*“The daily follow up calls were not inconvenient because just do it at the same time everyday, can expect it, show up, do it, get it off the plate and then carry on with the day.” (P4, Male, Age range 34-40, Unemployed, Interview 3)*

*“They’ve been really good. They’ve been pretty flexible in terms of the dates that you can do them on.” (P8, Agender, Age range 26-33, Unemployed, Interview 3)*

Researcher-participant miscommunication during scheduling was found to negatively impact participants’ willingness to participate. Returning to the IWK to complete the appointments requires participants to take time out of their day and should be as seamless as possible. When the follow up visits began inconveniencing participants, retention was affected, as explained by one participant who withdrew from the study for this reason:

*“…you have to wait outside the door for someone to let you in and nobody came for a half an hour and that was probably one of the main reasons that I stopped doing it because I had to take time out of my day to go do that… once I was back in real life, it was really annoying to have to physically go to the hospital and do all these things. I think it was just easier when I was in there for some reason. So that with the inconvenience of having to wait and not having someone there just made me, it didn’t feel worth it anymore.” (P10, Female, Age range 26-33, Employed fulltime, Interview 3)*

In terms of the sample collections involved during the follow up visits, it was found to be especially important that participants were adequately prepared on what to expect. Some participants reported being surprised upon arrival to the IWK to discover that they would be required to provide additional samples, making the appointments longer than initially anticipated.

*“Yes, there were [sample collections], which I didn’t actually expect. I thought it was just going to be physical, but they needed to do more blood work and everything else too. It wasn’t a problem, but I didn’t expect that.” (P14, Male, Age range 34-40, Employed part-time, Interview 2)*

Some participants reported having lost their skills in terms of providing sample collections upon returning to the IWK for follow up visits. The repetitiveness of the procedures during the inpatient stay helped desensitize participants to any discomfort; conversely, the prolonged time between outpatient visits was found to “re-sensitize” participants to the discomfort. Reliance on staff for emotional support and clear instructions was noted as important by some participants when providing samples during follow up visits.

*“I kind of lost my skill in how to do them between some of the follow up visits. Like when you’re in there and you’re doing them everyday it’s like alright, I’ve got the technique down. I figured it out. I’m not going to spray water all over the nurse today and then you come out and you don’t do it for a few weeks and then you’re like, how do I do the nasopharyngeal aspirate?” (P13, Female, Age range 18-25, Student, Unemployed, Interview 3)*

Delays in compensation payouts were noted as challenges for some participants who were relying on the income. Many were under the assumption that compensation would be provided immediately upon discharge. Those who planned to use the compensation towards immediate expenses were surprised by having to wait weeks to receive payment, attributing these issues to miscommunication.

*“…I had some problems with was receiving my compensation. It took like a month and a half… It was supposed to be a direct deposit, I gave them my banking information, but it ended up being a cheque which took even longer. So, because I wasn’t working at the time I was really relying on that money at the time, so that was a bit of an issue.” (P15, Female, Age range 26-33, Unemployed, Interview 3)*

#### Lasting impacts.

Although the *B. pertussis* infection itself did not pose any lasting physical health consequences on participants once antibiotic therapy had been completed, participation in the CHIM trial was found to have lasting impacts, both positive and negative. Physically, repetitive blood draws were noted to be painful and was found to impact participants’ day-to-day lives in the months following the inpatient period, as noted by one participant:

*“I was shifting off between arms between different draws, but as a right-handed person I’m using my right arm day to day, not so much my left so once I got out and started going to the gym and being active again, I noticed some tightness when fully extending my left arm… I feel like that’s probably something that if I had considered it prior to going in, doing regular stretches, making sure that I’m getting full range of motion… just a matter of breaking in the scar tissue or breaking in the healed tissue which I wasn’t properly doing when I was on site.” (P16, Male, Age range 26-33, Employed fulltime, Interview 3)*

One participant made mention of experiencing adverse effects of antibiotic therapy in the weeks following discharge from the isolation unit, citing gastrointestinal upset for which they were recommended a probiotic supplement by their physician.

*“And my stomach was a total wreck from the antibiotic… Because that level of antibiotic, it kills your gut bacteria, right. So, and I have a bad stomach to start with, so that was a little brutal.” (P19, Female, Age range 34-40, Employed fulltime, Interview 3)*

Mentally, the transition from being isolated to regular life can be challenging for some participants. Some participants felt overwhelmed by the stark change in environment during the first few hours to days of being discharged.

*“…I had been in a quiet, artificially lit, pretty cold room for so long then I came outside and it was loud and it was bright and it was hot and it was just like I wasn’t anticipating it being overwhelming. It doesn’t feel like three weeks is enough to forget what being outside is like but just driving down Robie Street was crazy. There was so many people.” (P13, Female, Age range 18-25, Student, Unemployed, Interview 3)*

Conversely, some participants relished their newfound freedom upon discharge, appreciating things they had previously taken for granted.

*“…but the following days I found, I just had almost like a giddiness. I could walk downstairs if I wanted to or I could walk outside if I wanted to. I could eat whatever I wanted to and drink whatever I wanted to and that was nice. Stuff you take for granted.” (P7, Female, Age range 34-40, Employed fulltime, Interview 3)*

The financial compensation offered to participants was found to have significant positive impacts on their lives post-isolation. Participants reported using the money to pay off debts or take time off work, particularly for students who would have normally held summer jobs. One participant explained how the financial compensation provided for participation in the trial enabled them to have more financial freedom in terms of their work:

*“I ended up having some issues with my job like later in the year and was able to take a month off because I was like oh, I made this extra money from the study so it actually enabled me to make decisions into my life that gave me more freedom later on.” (P10, Female, Age range 26-33, Employed fulltime, Interview 3)*

Many participants reported positive, supportive interactions with the nursing staff who cared for them in the isolation unit. Follow-up visits were appreciated by participants as opportunities to connect with staff, which made the visits more comfortable and alleviated any discomfort associated with sample collection. Participants valued the compassionate and person-centered approach of the nursing staff during their participation in the trial.

*“I think it’s really great that, even after the amount of people that they’ve had in there that the staff takes the time to memorize like little jokes we talked about or like the very first day I went I was wearing an Elton John t-shirt and then one of the staff would just bring up Elton John things even like 84 days later.” (P13, Female, Age range 18-25, Student, Unemployed, Interview 3)*

The positive lasting impacts of the trial were reflected in participants’ willingness to share their experiences with others post-isolation and recommend the trial to their friends and family. Many participants expressed having referred interested friends to the trial, some of whom ended up being eligible and enrolling. Overall, participants enjoyed being able to talk about their experiences and educate others about what it means to be a CHIM trial participant.

*“Pretty much anytime I ran into any friends socially after coming out of that there were a few questions coming from them about what the experience was like, what, did I get sick, was I symptomatic, how bad were the tests… it’s an interesting topic of conversation that way.” (P16, Male, Age range 26-33, Employed fulltime, Interview 3)*

*“…I think if it is somebody who feels like they can do it, I would recommend it, yeah.” (P20, Male, Age range 26-33, Employed fulltime, Interview 2)*

## Discussion

This study used semi-structured interviews among CHIM trial participants to develop a process-based emerging theoretical model grounded in the data. Our longitudinal approach allowed us to explore multiple aspects of the CHIM trial participant experience over time to obtain a holistic understanding of these valuable perspectives, expanding on prior survey-based [10,26] and cross-sectional research [11–13,27] into this area. We found the CHIM trial participant decision-making process and experience to be dependent on a mutually beneficial researcher-participant partnership rooted in trust. Three phases of the trusting partnership were identified across the course of the CHIM trial participant experience: (1) establishing a trusting partnership, (2) supporting a trusting partnership, and (3) maintaining a trusting partnership. Quality improvement (QI) recommendations for future CHIM trials were identified for each phase based on our findings.

Trust has been outlined as an integral precursor to the development and implementation of CHIM trials in public engagement studies [28,29]. In a public involvement consultation group for a cutaneous leishmaniasis CHIM study in the UK, participants spoke to the importance of providing clear recruitment materials with accurate study information related to the risk of infection itself to help prospective participants make an informed decision [28]. Our results echo these findings, with participants relying on accessible and accurate study information to establish trust in the researchers during the decision-making phase. For most participants, the CCfV’s website was the main source of reliable and accessible information during the decision-making phase. Other valuable sources of information included anecdotal reports from past participants. Mtunthama et al. [30] reported similar findings from their qualitative study of Malawian CHIM participants, explaining how involving former participants as advocates could help dispel any misconceptions about CHIM trials and subsequently improve recruitment.

In Chi et al.’s [27] qualitative study on the experiences of participants in a Kenyan malaria CHIM trial, participants reported doubts in the truthfulness, motivation, and professional standards of the researchers, stemming from a lack of trust in the novelty of CHIM trials. Preparation of the malaria challenge outside of participants’ view elicited skepticism of the injection itself for some participants, believing it to be contaminated with another pathogen. Our study participants did not report this degree of skepticism and generally reported a high level of trust in the safety and legitimacy of the researchers despite the novelty of CHIM trials, along with a consensus that such research would not be approved within Canada without it being ethically sound. Interestingly, another qualitative study based on the same Kenyan malaria CHIM trial reported high levels of trust in the researchers as an important factor in participants’ initial decision-making process, stemming from the research institution’s long-standing presence and positive relations within the community and a belief that they would not intentionally harm the community [31]. This suggests that, while trust is an important precursor to the initial decision-making process in all contexts, it is not always upheld throughout the CHIM trial participant experience. Our study identified factors that serve to support and maintain researcher-participant trust beyond the initial decision-making process.

We found the opinions of others to have an influence on the decision-making process of CHIM trial participants. This reflects some of what has already been reported in the literature. For example, Gbesmete et al. [29] found that potential participants in a SARS-CoV-2 CHIM trial noted the importance of including family, friends, and their employers in their decision. Similarly, one participant in a pneumococcal challenge study in Malawi felt that consulting their family was an important step in the decision-making process, serving to provide reassurance that a sensible decision was made [30]. Interestingly, some participants in our sample reported concealing participation from their workplace, which was found to be anxiety-inducing. In Njue et al.’s [13] qualitative study on participants in a Kenyan malaria CHIM trial, some participants reported concealing participation from their significant others due to concerns related to the novelty of the research and fear of stigmatization. Fear of stigmatization was especially apparent in female participants who tend to bear greater responsibility for day-to-day family and childcare in this area, leading to potential family disagreements. Similarly, Chi et al. [27] found that women who left children at home had a harder time being away from family during the inpatient portion of the study than their male counterparts. Participants in our study did not share these fears, with participants of all genders reporting inclusion of significant others in their decision-making process. While the delegation of household responsibilities was discussed as a logistical consideration of participation, no gender discrepancies were noted. This may reflect the impact of cultural differences between Canada and Kenya on CHIM trial participants, where patrilineal values in certain regions may dictate the decision-making process in women [27]. Not all participants in our study saw the opinions of others to have a strong influence on their decision-making process, citing a desire to participate despite receiving hesitancy or skepticism from family and friends. Similarly, Hoogerwerf et al.’s [12] quantitative survey of CHIM trial participant motivations found that 93% of respondents would participate regardless of the opinions of others. Interestingly, while Chi et al. [27] found that participants tended to cite the benefits of participation to convince skeptical family members and friends, namely ancillary care and financial compensation, participants in our study tended to reiterate the study’s safety.

We found the decision-making process to be multifactorial, involving careful consideration of both the risks of participation alongside the anticipated benefits. Mixed motivations have been reported by participants in various other types of research [9]; our results support this claim in the context of participation in CHIM trials. Our findings expand upon those of Oguti et al. [26] in which their use of descriptive survey methods determined a wide variety of motivations, both altruistic and financial, for participation in typhoid and paratyphoid CHIM studies in the UK. Their study identified the desire to contribute to the progress of medicine as the most frequently cited motivator, with financial compensation noted as a necessary pre-requisite for most participants, while acknowledging that the decision to partake in CHIM trials is influenced by a variety of economic and sociodemographic factors not captured through survey methods. Our findings addressed this knowledge gap by employing in-depth participant-led interviews to assess the meaning participants attribute to each of these risks and benefits.

While not every participant identified altruistic motivations as the primary driver of their decision, all participants acknowledged the societal benefits of participating in clinical research. We found that the perceived altruistic benefits of participation were augmented when participants had a close connection to whooping cough or other childhood respiratory illnesses. Similarly, individuals who come from malaria-endemic regions have been found to be more likely to cite altruistic motivations for participating malaria CHIM studies under the assumption that the outcomes of the study would be of benefit in the future to their own communities [31]. This suggests that proximity of the disease under study to oneself can serve to increase the perceived societal benefits of participation in a CHIM study, similar to findings by Cattapan et al. [32] where a personal or community connection to influenza was found to impact participant motivations in a phase 1 adjuvanted influenza vaccine trial. Furthermore, many participants in our study sample exhibited previous altruistic behaviours, such as blood donation, prior research participation, or surrogacy. In their survey on the motivations of potential volunteers in SARS-CoV-2 CHIM trials, Marsh et al. [10] reported a similar pattern, with participants demonstrating high levels of prior engagement in various forms of altruism.

While offering financial incentives for research participation has been scrutinized due to concerns related to undue influence [33], our findings did not indicate that financially motivated participants negated the risks of participation. By contrast, Stunkel and Grady [9] found financial motivations to be associated with a greater attention to risk. Our study supports this claim, as all participants reported careful consideration of the potential risks of study involvement regardless of motivations. Similarly, survey responses of individuals interested in a SARS-CoV-2 CHIM trial indicated consistently altruistic motivations without any special indication of poor risk perception or economic vulnerability [10]. One participant in our sample even made mention to appreciating the trial’s eligibility criteria of having a valid household address, thus avoiding the exploitation of persons experiencing homelessness who may be unduly influenced and strengthening their trust in the researchers. Other demographic or socioeconomic groups who have been found to be most vulnerable to exploitation include ethnic minorities, low-income earners, or unemployed individuals [10]. While participants in our sample had a range of annual household incomes, none displayed high levels of economic vulnerability such that the risks of participation were negated. Even those participants who self-disclosed financial hardships still appreciated the potential risks, as well as the societal benefits, of their participation.

The amount of compensation offered was overall well received by participants, with claims of the reimbursement being fair for the time commitment required. Some participants raised concerns regarding the day-by-day nature of the compensation, suggesting this may threaten the integrity of the study; similarly, Chi et al. [34] reported a competition emerging among participants in a Kenyan malaria challenge study to “make it to the end” by concealing symptoms to earn more money. While it is unlikely that participants could successfully deceive the researchers in this way due to frequent blood monitoring and clinical testing, it is worthwhile to consider the implications of different reimbursement strategies on recruitment when planning future CHIM trials.

Participants reported intentions to use the compensation towards paying off debts, investing, or, for students, supplementing summer employment. Some have suggested participants’ individual economic circumstances are more integral to the CHIM trial participant decision-making process than the absolute amount of compensation provided [26]. Our findings support this claim, with many participants citing their current financial, employment, or student status as justification for financial motivations. One participant in particular reporting a shift in motivations away from financial compensation upon obtaining a higher paying job. It should be reiterated that, despite this finding, we did not find evidence to support that those participants of lower economic status were unduly influenced.

While financial benefits were highlighted by our study sample as a necessity for some and a bonus for others, additional personal benefits were also highlighted, including access to full medical examinations during study screening. This was particularly beneficial for participants who were without family doctors, which is a rising concern for Nova Scotians [35]. Similarly, CHIM trial participants in Kenya cited accessing healthcare services, such as ECGs and liver function tests, as a motivating influence, given that these tests are expensive and often unavailable in public health care facilities [13]. Mtunthama et al. [30] also reported a desire to know one’s general health status, including HIV status, as a motivating factor for participants in a Malawi pneumococcal CHIM trial. Some participants in another Kenyan malaria CHIM trial perceived the health screening as more important than the financial benefits of participation [27]. Participants in our study did not identify accessing these healthcare services as being a primary motivating factor; rather, it was considered an additional bonus encountered during the experience. Similarly, accessing health care professionals was found to be an infrequently cited motivator for Nova Scotians participating in phase 1 vaccine trials with the CCfV [32]. Our findings related to accessing health screenings are therefore unsurprising, given that participants for the CHIM study were recruited from the same population as the abovementioned study and reflect the Nova Scotian context (i.e., free healthcare). As the percentage of Nova Scotians without family doctors continues to increase and accessing health screenings becomes less accessible, it is possible that future CHIM trial participants’ motivations may shift to reflect this.

Other personal benefits of participation identified by participants in our sample included using the time in isolation as a vacation and having new experiences. Many participants were intrigued by the novel nature of the CHIM trial, citing the recruitment tagline of, “Are you up for the challenge?” as being enticing, evoking a sense of adventure and offering an opportunity for personal growth. Similarly, Hoogerwerf et al. [12] identified curiosity as a motivation for CHIM trial participation, appreciating having had the experience of going through a trial with the other participants and the study team. Novel or high-profile research has been identified as a potential incentive for clinical trial participation, as was the case for the CCfV’s 2015 phase 1 Ebola vaccine trial [32].

For CHIM trials to be ethically permissible, they must minimize risk to participants [4]. Minimal risk for participants in non-therapeutic clinical trials has been defined as risks that would expose individuals to no greater likelihood of harm than those encountered in everyday life [36]. This can be achieved through rigorous ethical review, appropriate study design, employing trained personnel, providing appropriate compensation, maximizing the social value of the research, and ensuring participants understand the risks and benefits [37]. Participants in our sample appreciated the ongoing consent sought each day of the inpatient isolation stay, acknowledged the altruistic benefits of participation regardless of motivation, and overall displayed a thorough understanding of the risks of *B. pertussis* infection, demonstrating an acceptable and minimal risk level. Strong participant-researcher trust is integral in facilitating an environment which minimizes risk level (i.e., enhancing participants’ understanding and appreciation of the risks involved).

The consensus in our study sample was that the physical health risk of whooping cough to healthy adult volunteers was relatively low, given that whooping cough primarily affects young infants and the elderly, demonstrating a strong sense of trust in the research methods and procedures. Furthermore, many of our participants reported high levels of confidence that they would not become seriously ill from *B. pertussis* inoculation due to self-reported “strong immune systems”. Healthy volunteers in clinical trials have been found to distinguish the overall risk of participation from their own personal risk, believing that even if a clinical trial in general was risky, they themselves were unlikely to be harmed [38]. Stunkel and Grady [9] found the medical risk of a clinical trial to be the main disincentive to participation; as our study did not capture the perspectives of individuals who elected not to participate, future research into the experiences of CHIM trial participants and non-participants’ decision-making processes would help illustrate factors contributing to enrollment.

Beyond the physical health risks, many of our participants also believed they would be immune to the mental health risks associated with isolation, despite acknowledging the potential for negative psychological effects. A systematic review of patients’ experiences living in isolation found the physical process of isolation and related sense of uncertainty and loss of control to have a negative impact on patients’ moods, including increased rates of depression, anxiety, fear, and hostility [39]. Participants in our sample implemented effective coping strategies throughout isolation, including bringing in personal items from home to familiarize the space and consistent virtual communication with family and friends. Participants attributed their preparedness for the inpatient isolation stay to prior isolation experience from Covid-19, the attentiveness of the study staff, and the constant availability of social supports, including the Discord server and the nursing staff. Although not all participants took advantage of these supports, there was a widespread appreciation towards their potential, serving to support the trusting researcher-participant relationships. The importance of psychological supports for isolated CHIM trial participants has been highlighted in public engagement work, with suggestions including the employment of psychologists or the provision of online participant support networks [29]. Our findings support this claim, reiterating the importance of acknowledging and preparing participants for the potential psychological risks associated with CHIM trial participation and providing accessible supports. For instance, while the Discord server was identified as a valuable resource to connect with other participants and ease the burden of isolation, issues with connectivity and access were identified as barriers to achieving its potential.

The physical environment of the isolation rooms was found to have a significant impact on participants’ experiences, affecting the way in which they were able to work, live, and relax in the space. The overall response to the space was positive; however, participants did identify certain aspects of the hospital environment which proved to be challenging throughout their experience, including uncontrollable noises, distracting lights, unaesthetically pleasing medical devices, and sometimes unpleasant hospital food. These unexpected challenges sometimes hindered participants’ trust in the researchers; some participants reported feeling deceived by the researchers upon discovering these negative aspects of the physical space. Towards the end of the inpatient isolation stay, many participants were anxious to return to the familiarity of their homes. By contrast, Chi et al.’s [27] found CHIM trial participants in Kenya to describe the positive residential experiences in isolation as an incentive to stay in the study longer, appreciating the environment, amenities, and access to good food and entertainment. It should be noted that residency in the beforementioned study did not involve single-room isolation units as ours did; rather, participants were able to interact face-to-face with other participants in their cohorts. Despite the uncontrollable nature of many aspects of the physical environment, enhanced participant education prior to the inpatient stay may serve to better prepare participants for the isolation experience to support the trusting researcher-participant partnership, as suggested by both Knowles [40] and the participants in our study sample. Additionally, careful monitoring of participant experiences throughout CHIM trials through responsive research designs is paramount in addressing such unanticipated burdens [34]. Participants cited the responsive nature of the trial staff as an important facilitator in supporting the researcher-participant partnership. Some burdens, such as poor Internet access, limited availability of dietary-friendly snacks, or unpleasant noises from the unit, that participants reported during initial interviews had oftentimes been addressed upon subsequent interviews because of the attentiveness of staff.

Setting realistic expectations for the inpatient isolation stay was found to be critical to ensure researcher-participant trust is upheld throughout the duration of participation. We found strong researcher-participant communication to be integral in setting these expectations; likewise, miscommunication risked breaking participants’ trust in the researchers, leading to feelings of deception. For instance, some participants reported feeling surprised by the lack of privacy in the rooms, the quality and timing of the food service, and the level of dependency on the research staff required while in isolation. Early interactions with research teams beginning before consent processes play an important role in ensuring participants fully understand the study [41]. Kelley et al. [42] found that individuals with prior trusting relationships with members of the research team can strengthen these interactions. Some participants in our sample were familiar with members of the research team, citing this as a facilitator to their decision to enrol and aiding them in setting expectations for the experience. Strengthening communication and building interpersonal relationships with prospective participants early in the decision-making process may facilitate accurate expectations for the inpatient isolation stay, thus supporting the trusting researcher-participant partnership.

Participant retention in clinical trials is critical to uphold the study’s validity and avoid any potential costs of participant withdrawal [43]. Upon completing the inpatient isolation phase of the CHIM trial, participants must remain motivated to facilitate participant retention on an outpatient basis. According to self-determination theory, human motivation can be differentiated between controlled motivation, which depends on rewards or punishments, and autonomous motivation, which involves behaving with a sense of volition, agency, and choice [44]. Autonomous motivation is preferred in clinical trial settings; autonomously motivated participants are more wholeheartedly engaged than those who are controlled in their motivations [45]. Strategies to support participants’ autonomous motivation in clinical trials include tailoring elements of the study experience to suit participants’ needs, offering flexibility, and being accommodating to participants throughout the trial [43]. Most of the participants in our sample remained enrolled throughout the entire year-long duration of the CHIM trial and appreciated the flexibility of the outpatient visits and the staff’s accommodating nature. We found the convenience of the visits to be a key factor in sustaining participants’ motivation on an outpatient basis, along with strong researcher-participant communication. Conversely, miscommunication hindered trust in the researchers and resulted in study withdrawal for one participant in our sample; when the follow up visits became an inconvenience, they displayed a lack of autonomous motivation, stating that participation no longer felt “worth it”.

The findings of our study expand upon the existing literature on the perspectives of CHIM trial participants, contextualizing the complex motivations involved in the decision-making process while identifying aspects of the experience that may be challenging or lacking. From our results, we were able to identify some of the logistical issues faced by CHIM trial participants and offer pragmatic solutions for the consideration of researchers designing and planning future CHIM trials. These QI recommendations (Table 2) can assist in establishing, supporting, and maintaining a trusting researcher-participant partnership and subsequently improve recruitment and retention.

**Table 2:** QI Recommendations.

*Establishing*	
*Informative recruitment messaging*	CHIM trial recruitment messaging should focus on providing clear and concise information about the study’s intent and methods, while highlighting the nature of participation and what can be expected, so the public can assess their understanding of the study and desire to become a participant. CHIM trial recruitment messaging should highlight certain aspects of the study which may be enticing/motivational for some participants, beyond financial compensation.
*Accessible online information*	Researchers should use visuals when providing online information about CHIM trial participation to help potential participants set realistic expectations about their stay (e.g., Video tours of the isolation unit, photos of the staff). Online information related to the *B. pertussis* challenge should be provided using lay language to ensure participants understand why the duration of the isolation stay is subject to change, allowing them to set realistic expectations and plan accordingly.
*Partnerships with former participant advocates*	Past participants in CHIM trials can provide valuable sources of information for potential participants by sharing their experiences and providing insight into the nature of participation. Researchers should consider collaborating with past participants to improve recruitment and participant advocacy.
*Comprehensive screening*	Participants should be given ample time to ask questions and tour the unit during initial screening visits to facilitate realistic expectations and strengthen participants’ preparedness prior to their challenge dates. All pertinent information related to the CHIM trial procedures (e.g., Variable duration of stay) should be relayed to participants in person, and participant understanding should be assessed.
***Supporting***	
*Physical environment*	Careful consideration of the participant-facing impacts of the physical space when designing CHIM trials can assist researchers in supporting the relationships they have established with the trial participants (e.g., Will participants be disturbed in this space when trying to sleep, work, or relax? What does the unit look like? Sound like? Feel like?). Providing tours of the isolation unit to participants prior to their challenge date can facilitate accurate expectations and allow participants to plan accordingly when packing and preparing for their stay.
*Sample collection*	Study staff should learn and accommodate, to the best of their ability, participants’ individual schedules and preferences into daily trial needs (e.g., Tailoring sample collection to individual work schedules).
*Participant education*	Providing sufficient written material detailing all study procedures in clear, accessible language, with visuals as appropriate, for participant referral throughout the CHIM trial and assessing and addressing any knowledge gaps early on can aide researchers in supporting their relationships with participants (e.g., Do participants know how to provide a urine sample? Have they ever seen an NPA tube? Do they understand why the duration of stay is subject to change?)
***Maintaining***	
*Follow up visits*	Instructions for follow up visits should be clear and easy to follow and remain consistent throughout participation. Study staff should ensure participants are adequately prepared for what is to be expected (e.g., Do participants know what samples they will provide during follow ups? Do they know how much time appointments will take?) Follow up visits should be scheduled at the participants’ convenience, whenever possible.
*Discharge information*	Discharge instructions should include information surrounding potential lasting impacts from study procedures (e.g., Do participants know the possible adverse effects of their antibiotic therapy? Can they expect to have any residual soreness from the sample collection? Do they have any concerns about the transition from isolation back into regular life?)
*Compensation*	Realistic timelines for compensation payouts should be provided to participants to minimize any financial stressors on participants.

## Limitations

This study had limitations which should be considered when interpreting our findings. Firstly, issues with recruitment and retention affected our sample size. Passive recruitment for the sub-study using flyers distributed by the Challenge Unit team proved to be ineffective; rather, actively recruiting participants face to face on the Challenge Unit produced higher levels of interest. Future research into this area should consider this active recruitment approach to capture all interested CHIM trial participants in their samples. Participant retention throughout the longitudinal approach to this study also proved challenging, as many participants failed to respond to email reminders for the third and fourth time point interviews once they were discharged from the Challenge Unit. We attributed this to scheduling conflicts with participants’ lives and responsibilities that were less challenging to navigate when participants were living in isolation on the Challenge Unit. Although not all participants were interviewed four times for this reason, and despite recruiting less participants than initially anticipated, we did reach consensus that saturation was obtained. This resulted in the termination of the fourth interviews towards the end of data collection and analysis as no new or relevant information was ascertained from these final interviews. Participants were, however, encouraged to reach out should any aspect of their experience change or if they had new information which they wished to have included in the study. Some participants felt they could express themselves better in written formats than oral. Future research into this area could consider other ways to gather participant data in addition to in-depth interviews.

As stated previously, this study did not capture the perspectives or experiences of potential participants who elected not to participate or those who did not meet the CHIM trial eligibility criteria and were screened out. These perspectives may have enriched our findings by providing a more nuanced understanding of the CHIM trial non-participant experience. Future research may benefit from seeking these perspectives and integrating them into QI recommendations.

Finally, our study sample was predominantly white, displaying a lack of ethnic diversity. A representative and diverse sample would ensure perspectives from non-Western cultures are captured and reflected in the findings. It is also important to note that findings from a single sample may not be transferrable to other settings.

## Conclusion

This study addressed a significant knowledge gap in the literature surrounding the decision-making processes and experiences of CHIM trial participants. Our modified grounded theory study used semi-structured interviews with participants enrolled in CCfV’s *B. pertussis* CHIM trial to develop an emergent conceptual model depicting the CHIM trial participant experience over time. The core category that emerged from this process was “A Trusting Partnership”, describing the dynamic researcher-participant relationship throughout CHIM trial participation. Our model spanned three phases of CHIM trial participation: the decision-making phase, the inpatient isolation phase, and the outpatient phase. Across all three phases, effective researcher-participant communication is paramount in shaping the participant experience. Factors that serve to strengthen or hinder the trusting researcher-participant partnership were identified across all phases. Subsequently, QI recommendations were identified across the emergent conceptual model for the consideration of researchers, decision-makers, and clinicians who are planning and coordinating future CHIM trials. These recommendations are valuable in that they can serve to improve the experiences of CHIM trial participants, subsequently improving recruitment and retention. Future research should assess the feasibility and impact of implementing these recommendations in practice in other types of CHIM trials and settings.

## Supporting information

S1 File Attachments. **Attachment 1**-Guide for 1^st^ and 2^nd^ interviews. **Attachment 2**-Guide for 3^rd^ and 4^th^ interviews.
(DOCX)

## References

[1] Darton TC, Blohmke CJ, Moorthy VS, Altmann DM, Hayden FG, Clutterbuck EA, et al. Design, recruitment, and microbiological considerations in human challenge studies. Lancet Infect Dis. 2015;15(7):840–51. doi: 10.1016/S1473-3099(15)00068-7 26026195

[2] Sauerwein RW, Roestenberg M, Moorthy VS. Experimental human challenge infections can accelerate clinical malaria vaccine development. Nat Rev Immunol. 2011;11(1):57–64. doi: 10.1038/nri2902 21179119

[3] World Health Organization. WHO guidance on the ethical conduct of controlled human infection studies. Geneva: World Health Organization. 2021.

[4] Miller FG, Grady C. The ethical challenge of infection-inducing challenge experiments. Clin Infect Dis. 2001;33(7):1028–33. doi: 10.1086/322664 11528576

[5] Kestelyn E, Le Phuong C, Ilo Van Nuil J, Dong Thi HT, Minh Nguyen N, Dinh The T, et al. Expert voices and equal partnerships: establishing Controlled Human Infection Models (CHIMs) in Vietnam. Wellcome Open Res. 2019;4:143. doi: 10.12688/wellcomeopenres.15337.1 31681857 PMC6820821

[6] Lemmens T, Elliott C. Guinea pigs on the payroll: the ethics of paying research subjects. Account Res. 1999;7(1):3–20. doi: 10.1080/08989629908573939 11657561

[7] Tishler CL, Bartholomae S. The Recruitment of Normal Healthy Volunteers: A Review of The Literature on the Use of Financial Incentives. The Journal of Clinical Pharma. 2002;42(4):365–75. doi: 10.1177/00912700222011409 11936560

[8] Elliot C. Guinea-pigging: Healthy human subjects for drug-safety trials are in demand. But is it a living?. The New Yorker. 2008;36–41. 18265451

[9] Stunkel L, Grady C. More than the money: a review of the literature examining healthy volunteer motivations. Contemp Clin Trials. 2011;32(3):342–52. doi: 10.1016/j.cct.2010.12.003 21146635 PMC4943215

[10] Marsh AA, Magalhaes M, Peeler M, Rose SM, Darton TC, Eyal N, et al. Characterizing altruistic motivation in potential volunteers for SARS-CoV-2 challenge trials. PLoS ONE. 2022;17(11):e0275823. doi: 10.1371/journal.pone.0275823 PMC9629635 36322529

[11] Kraft SA, Duenas DM, Kublin JG, Shipman KJ, Murphy SC, Shah SK. Exploring Ethical Concerns About Human Challenge Studies: A Qualitative Study of Controlled Human Malaria Infection Study Participants’ Motivations and Attitudes. J Empir Res Hum Res Ethics. 2019;14(1):49–60. doi: 10.1177/1556264618820219 30585505

[12] Hoogerwerf M-A, de Vries M, Roestenberg M. Money-oriented risk-takers or deliberate decision-makers: a cross-sectional survey study of participants in controlled human infection trials. BMJ Open. 2020;10(7):e033796. doi: 10.1136/bmjopen-2019-033796 32713843 PMC7383945

[13] Njue M, Njuguna P, Kapulu MC, Sanga G, Bejon P, Marsh V, et al. Ethical considerations in Controlled Human Malaria Infection studies in low resource settings: Experiences and perceptions of study participants in a malaria Challenge study in Kenya. Wellcome Open Res. 2018;3:39. doi: 10.12688/wellcomeopenres.14439.2 29806038 PMC5954342

[14] Phillippi J, Lauderdale J. A Guide to Field Notes for Qualitative Research: Context and Conversation. Qual Health Res. 2018;28(3):381–8. doi: 10.1177/1049732317697102 29298584

[15] Kilgore PE, Salim AM, Zervos MJ, Schmitt H-J. Pertussis: Microbiology, Disease, Treatment, and Prevention. Clin Microbiol Rev. 2016;29(3):449–86. doi: 10.1128/CMR.00083-15 27029594 PMC4861987

[16] Glaser B, Strauss A. The discovery of grounded theory. New York: Aldine Publishing Company; 1967.

[17] Morgan DL. Pragmatism as a Paradigm for Social Research. Qualitative Inquiry. 2014;20(8):1045–53. doi: 10.1177/1077800413513733

[18] Chun Tie Y, Birks M, Francis K. Grounded theory research: A design framework for novice researchers. SAGE Open Med. 2019;7:2050312118822927. doi: 10.1177/2050312118822927 30637106 PMC6318722

[19] IWK Health [Internet]. Patient and Family-Centered Care. Available from: http://www.iwk.nshealth.ca/pfcc

[20] Johnson JL, Adkins D, Chauvin S. A Review of the Quality Indicators of Rigor in Qualitative Research. Am J Pharm Educ. 2020;84(1):7120. doi: 10.5688/ajpe7120 32292186 PMC7055404

[21] Conlon C, Timonen V, Elliott-O’Dare C, O’Keeffe S, Foley G. Confused About Theoretical Sampling? Engaging Theoretical Sampling in Diverse Grounded Theory Studies. Qual Health Res. 2020;30(6):947–59. doi: 10.1177/1049732319899139 31959073

[22] Birks M, Chapman Y, Francis K. Memoing in qualitative research. Journal of Research in Nursing. 2008;13(1):68–75. doi: 10.1177/1744987107081254 PMC12602296 41229605

[23] Hadi MA, José Closs S. Ensuring rigour and trustworthiness of qualitative research in clinical pharmacy. Int J Clin Pharm. 2015. doi: 10.1007/s11096-015-0237-6 26666909

[24] Strauss A, Corbin JM. Basics of qualitative research: Grounded theory procedures and techniques. Sage Publications, Inc. 1990.

[25] Street RL Jr, Makoul G, Arora NK, Epstein RM. How does communication heal? Pathways linking clinician-patient communication to health outcomes. Patient Educ Couns. 2009;74(3):295–301. doi: 10.1016/j.pec.2008.11.015 19150199

[26] Oguti B, Gibani M, Darlow C, Waddington CS, Jin C, Plested E, et al. Factors influencing participation in controlled human infection models: a pooled analysis from six enteric fever studies. Wellcome Open Res. 2019;4:153. doi: 10.12688/wellcomeopenres.15469.1

[27] Chi PC, Owino EA, Jao I, Olewe F, Ogutu B, Bejon P, et al. Understanding the benefits and burdens associated with a malaria human infection study in Kenya: experiences of study volunteers and other stakeholders. Trials. 2021;22(1):494. doi: 10.1186/s13063-021-05455-7 34311781 PMC8313115

[28] Parkash V, Jones G, Martin N, Steigmann M, Greensted E, Kaye P, et al. Assessing public perception of a sand fly biting study on the pathway to a controlled human infection model for cutaneous leishmaniasis. Res Involv Engagem. 2021;7(1):33. doi: 10.1186/s40900-021-00277-y 34053461 PMC8164890

[29] Gbesemete D, Barker M, Lawrence WT, Watson D, de Graaf H, Read RC. Exploring the acceptability of controlled human infection with SARSCoV2-a public consultation. BMC Med. 2020;18(1):209. doi: 10.1186/s12916-020-01670-2 32635912 PMC7339437

[30] Mtunthama Toto N, Gooding K, Kapumba BM, Jambo K, Rylance J, Burr S, et al. “At first, I was very afraid”-a qualitative description of participants’ views and experiences in the first Human Infection Study in Malawi. Wellcome Open Res. 2021;6:89. doi: 10.12688/wellcomeopenres.16587.2 35187267 PMC8825950

[31] Jao I, Marsh V, Che Chi P, Kapulu M, Hamaluba M, Molyneux S, et al. Deliberately infecting healthy volunteers with malaria parasites: Perceptions and experiences of participants and other stakeholders in a Kenyan‐based malaria infection study. Bioethics. 2020;34(8):819–32. doi: 10.1111/bioe.12781 32643809 PMC7689838

[32] Cattapan A, Browne K, Halperin DM, Di Castri A, Fullsack P, Graham J, et al. Motivation for participating in phase 1 vaccine trials: Comparison of an influenza and an Ebola randomized controlled trial. Vaccine. 2019;37(2):289–95. doi: 10.1016/j.vaccine.2018.11.014 30528592

[33] Dickert N, Grady C. What’s the price of a research subject? Approaches to payment for research participation. N Engl J Med. 1999;341(3):198–203. doi: 10.1056/NEJM199907153410312 10403861

[34] Chi PC, Owino EA, Jao I, Bejon P, Kapulu M, Marsh V, et al. Ethical considerations around volunteer payments in a malaria human infection study in Kenya: an embedded empirical ethics study. BMC Med Ethics. 2022;23(1):46. doi: 10.1186/s12910-022-00783-y 35443642 PMC9019790

[35] Ettinger L. People waiting for a family doctor in Nova Scotia could have years without finding one. CBC News. 2024 Mar 12. Available from: https://www.cbc.ca/news/canada/nova-scotia/family-practice-registry-record-number-1.7140349

[36] Evers DL, Fowler CB, Mason JT, Mimnall RK. Deliberate Microbial Infection Research Reveals Limitations to Current Safety Protections of Healthy Human Subjects. Sci Eng Ethics. 2015;21(4):1049–64. doi: 10.1007/s11948-014-9579-z 25150847

[37] Hope T, McMillan J. Challenge studies of human volunteers: ethical issues. J Med Ethics. 2004;30(1):110–6. doi: 10.1136/jme.2003.004440 14872087 PMC1757139

[38] Fisher JA, McManus L, Cottingham MD, Kalbaugh JM, Wood MM, Monahan T, et al. Healthy volunteers’ perceptions of risk in US Phase I clinical trials: A mixed-methods study. PLoS Med. 2018;15(11):e1002698. doi: 10.1371/journal.pmed.1002698 30457992 PMC6245523

[39] Abad C, Fearday A, Safdar N. Adverse effects of isolation in hospitalised patients: a systematic review. J Hosp Infect. 2010;76(2):97–102. doi: 10.1016/j.jhin.2010.04.027 20619929 PMC7114657

[40] Knowles HE. The experience of infectious patients in isolation. Nurs Times. 1993;89(30):53–6. 8233859

[41] Kraft SA, Duenas DM, Lewis H, Shah SK. Bridging the Researcher-Participant Gap: A Research Agenda to Build Effective Research Relationships. Am J Bioeth. 2020;20(5):31–3. doi: 10.1080/15265161.2020.1745936 32364474 PMC7241299

[42] Kraft SA, Duenas DM, Lewis H, Shah SK. Bridging the Researcher-Participant Gap: A Research Agenda to Build Effective Research Relationships. Am J Bioeth. 2020;20(5):31–3. doi: 10.1080/15265161.2020.1745936 32364474 PMC7241299

[43] Gamble E, Linehan C, Heavin C. Establishing Requirements for Technology to Support Clinical Trial Retention: Systematic Scoping Review and Analysis Using Self-determination Theory. J Med Internet Res. 2023;25:e38159. doi: 10.2196/38159 37052985 PMC10141281

[44] Williams GC, Saizow RB, Ryan RM. The importance of self-determination theory for medical education. Acad Med. 1999;74(9):992–5. doi: 10.1097/00001888-199909000-00010 10498090

[45] Ryan RM, Patrick H, Deci EL, Williams GC. Facilitating health behaviour change and its maintenance: Interventions based on Self-Determination Theory. European Health Psychologist. 2008;10:1–4.

