# Supplementary material for: Exploring the participant experience in controlled human infection model (CHIM) trials: A modified grounded theory study
Source: PLoS One. 2025 Aug 6;20(8):e0328378. doi: 10.1371/journal.pone.0328378 (PMC12327663; doi:10.1371/journal.pone.0328378)
Supplement: S1 File — Attachment 1-Guide for 1st and 2nd interviews. Attachment 2-Guide for 3rd and 4th interviews. (DOCX) [file pone.0328378.s001.docx]

**Attachments**

**Attachment 1.** Guide for 1^st^ and 2^nd^ interviews

**Preamble:** *[read after completing verbal consent confirmation / after reviewing interviewee questions]* As mentioned, the point of our time today is to discuss your experience leading up to, and during your time as a trial participant.

*[Include one of the two following statements as applicable]* For the most part you will be able to direct the course of our discussion, although I will ask some clarifying questions **OR** We will start with a broad question which you can answer as you feel best. Then, I have questions I’d like to ask you. These questions reflect things we’ve heard so far from other interviewees.

**Broad questions:** *[Present interviewee with broad question corresponding to the interview they are completing]*

| 1st interview, broad question | Can you tell me about the steps that led you to enrolling in the trial, and your experience so far? |
| --- | --- |
| 2^nd^ interview, broad question | Now that you are roughly two weeks into the trial, can you tell me about your experience as a participant so far? |

*[Use clarifying questions as needed; examples include]*

- Can you tell me more about [topic]?
- What did [topic / observation / etc] contribute to your decision to enroll?
- Could you tell me more about what [topic / phrase / etc] means to you?
- You mentioned [topic] earlier; how did this contribute to your [decision-making / experience / etc]?

*[If applicable, include the following probes]*

**NOTE FOR REB REVIEW: probes will be adjusted by the interviewers prior to meeting with interviewees. Probes will reflect the needs of data analysis at the time of each interview. Examples are provided below to demonstrate the types of probes that may be presented to interviewees.**

- In speaking with other interviewees, we have heard that [topic / issue / etc] was an important consideration when deciding whether or not to enroll. Did you also consider this, and if so how did it contribute to your decision?
- We have heard different things regarding [part of trial enrollment, e.g. consent discussion]; some people felt it was clear, however others felt it was not. What were your thoughts on [enrollment / consent discussion]? In what ways was it clear or unclear?
- What about the trial have you found most surprising so far?
- [During 2^nd^ interview] Have your thoughts on the trial changed in any way since we last spoke?

**Closing:** [*Ask the interviewee if they have anything further to add, or if there are any important details they want to be sure we capture in our data.]*

**Attachment 2.** Guide for 3^rd^ and 4^th^ interviews

**Pre-interview preparation for each interviewee:**

*[3^rd^ interview: Share the current draft patient journey map with the interviewee at least 1 week before the scheduled date of their 3^rd^ interview.]*

*[4^th^ interview: At least 1 week before each interviewee’s 4^th^ interview, share their personal revised patient journey map].*

**Preamble:** *[First address any questions the interviewee may have regarding the study or interview, then read the preamble]* The point of this interview is to discuss with you some observations we’ve made during analysis so far. We would also like to discuss the ‘journey map’ we have developed based on our earlier conversations.

**Questions**

**NOTE FOR REB REVIEW: The exact questions presented to interviewees during 3^rd^ and 4^th^ interviews will depend on the status of data analysis, and on the aspects of their PJM they are most interested in discussing. The PJMs will also depend on the observations made during analysis (3^rd^ interview), and on each interviewee’s feedback on the draft map (4^th^ interview). The following questions are provided as examples of the types of things that will be discussed.**

Trial procedures post-isolation

- Is there anything you would like to say about the isolation period now that you’ve had a few weeks to think about it?
- Is there anything you would like to say about the follow-up appointments after your isolation period, or about other trial activities?
- *[NOTE probes may be added as analysis progresses, similar to interviews 1 and 2].*

Patient journey map

- Overall, what are your impressions of the map? Does it seem to accurately reflect your experience in the trial?
- Does the map make sense to you, or are there things you find unclear?
- Does anything in the map surprise you?
- Do you think the map is complete, or would you recommend further changes?
  - *[If changes are requested]* What changes would you suggest we make to the map, so that it better reflects your experience? This could include adding details, changing something, etc.

*[The interviewee and interviewer can make notes during discussion. These should be included in the data if possible. After the 4^th^ interview, the interviewee can request one final review of their individualized journey map.]*
